# Long-term quality of life in patients with acute respiratory distress syndrome requiring extracorporeal membrane oxygenation for refractory hypoxaemia

**DOI:** 10.1186/cc11811

**Published:** 2012-10-19

**Authors:** Carol L Hodgson, Kate Hayes, Tori Everard, Alistair Nichol, Andrew R Davies, Michael J Bailey, David V Tuxen, David J Cooper, Vin Pellegrino

**Affiliations:** 1 Physiotherapy Department, The Alfred Hospital, Commercial Rd, Prahran, 3181, Australia; 2 Australia and New Zealand Intensive Care-Research Centre, School of Public Health and Preventive Medicine, Monash University, 99 Commercial Rd, Melbourne, 3004, Australia; 3 Intensive Care Unit, The Alfred Hospital, Commercial Rd, Prahran, 3181, Australia; 4 The School of Medicine and Medical Sciences, University College Dublin, Dublin 4, Ireland

## Abstract

**Introduction:**

The purpose of the study was to assess the long term outcome and quality of life of patients with acute respiratory distress syndrome (ARDS) receiving extracorporeal membrane oxygenation (ECMO) for refractory hypoxemia.

**Methods:**

A retrospective observational study with prospective health related quality of life (HRQoL) assessment was conducted in ARDS patients who had ECMO as a rescue therapy for reversible refractory hypoxemia from January 2009 until April 2011 in a tertiary Australian centre. Survival and long-term quality of life assessment, using the Short-Form 36 (SF-36) and the EuroQol health related quality of life questionnaire (EQ5D) were assessed and compared to international data from other research groups.

**Results:**

Twenty-one patients (mean age 36.3 years) with ARDS receiving ECMO for refractory hypoxemia were studied. Eighteen (86%) patients were retrieved from external intensive care units (ICUs) by a dedicated ECMO retrieval team. Eleven (55%) had H1N1 influenza A-associated pneumonitis. Eighteen (86%) patients survived to hospital discharge. Of the 18 survivors, ten (56%) were discharged to other hospitals and 8 (44%) were discharged directly home. Sequelae and health related quality of life were evaluated for 15 of the 18 (71%) long-term survivors (assessment at median 8 months). Mean SF-36 scores were significantly lower across all domains compared to age and sex matched Australian norms. Mean SF-36 scores were lower (minimum important difference at least 5 points) than previously described ARDS survivors in the domains of general health, mental health, vitality and social function. One patient had long-term disability as a result of ICU acquired weakness. Only 26% of survivors had returned to previous work levels at the time of follow-up.

**Conclusions:**

This ARDS cohort had a high survival rate (86%) after use of ECMO support for reversible refractory hypoxemia. Long term survivors had similar physical health but decreased mental health, general health, vitality and social function compared to other ARDS survivors and an unexpectedly poor return to work.

## Introduction

Acute respiratory distress syndrome (ARDS) is an inflammatory condition of the lung parenchyma that can result in reduced health-related quality of life (HRQoL) up to 5 years after discharge from the ICU [1-5]. The Australian incidence of ARDS in the late 1990s, was reported to be 28 cases per 100,000 per annum, with a 32% (95% CI 25, 40%) mortality rate [6]. In about 10% of cases, despite optimal management patients developed sustained hypoxemia refractory to standard interventions and had a higher risk of mortality [7,8]. These patients frequently receive hypoxemic rescue therapies, such as recruitment maneuvers, inhaled nitric oxide, prone positioning, high frequency oscillatory ventilation or extra-corporeal membrane oxygenation (ECMO) [9-11].

Several studies have reported that ECMO may improve survival in severe ARDS, but it is potentially associated with serious complications [12-14] and there have been few studies to review long-term survival and quality of life of ARDS patients following ECMO support [15-18]. It is unclear from the current literature if patients with ARDS have increased risk of physical, mental or cognitive disability as a result of the use of ECMO for refractory hypoxaemia [18]. Given the worldwide expansion in the use of ECMO since the H1N1 influenza A pandemic in 2009, there is an urgent need to determine the long-term outcomes in survivors [19]. Decreased long-term quality of life after ECMO could potentially be due to the severity of the disease, the ECMO complications or both. In a recent study of ARDS survivors from France, survivors who received ECMO as a rescue therapy had lower HRQoL compared to a French age- and sex-matched general population at one year but not lower than age- and sex-matched survivors of severe ARDS who were not treated with ECMO [18].

Australian ICUs have previously demonstrated a favorable mortality profile associated with the early utilization of ECMO for refractory hypoxemia in ARDS associated with influenza [19]. However the quality of survival in this Australian patient group is unknown and therefore the aims of our study were as follows. In Australian patients who were previously well and receiving veno-venous (VV)-ECMO for severe hypoxemia, we aimed to 1) measure the complication rates and ICU outcomes associated with ECMO; 2) pilot endpoints that could potentially be measured in ECMO survivors by telephone if they were retrieved from remote and rural areas; 3) measure the long term quality of survival, and 4) compare the HRQoL of Australian ECMO survivors with published international data from other research groups, both with and without the use of ECMO [17,20], including one cohort followed up from the H1N1 pandemic [18]

Our hypothesis was that HRQoL would be reduced in survivors of ECMO as a result of the complications of ECMO and not as a result of the severity of illness. We aimed to address this by comparison with similar international cohorts with severe ARDS. This is important as treatment of patients with severe ARDS using ECMO in Australia may demonstrate a favorable mortality profile, but is an invasive and expensive rescue therapy that has yet to be proven in a randomized clinical trial [21]. If ECMO survivors did have reduced HRQoL compared to non-ECMO survivors, identification of the cause may enable clinicians to reduce the complication rate and improve HRQoL.

## Materials and methods

This study was approved by the Human Research Ethics Committee at The Alfred Hospital, Melbourne, Australia, which waived the need for informed consent for the retrospective collection of demographic, physiological and hospital outcome data. However, informed consent was sought from survivors for the prospective long-term assessment of HRQoL.

We studied patients with ARDS between January 2009 and April 2011 because this period was a time of high ECMO utilization due to the H1N1 pandemic. Patients were retrospectively identified from the institution's prospective ECMO database. We included all adult patients with a confirmed diagnosis of ARDS considered potentially reversible by the treating clinician [22]. Patients who were under 18 years of age or who had lung disease that was considered irreversible (for example, cystic fibrosis) were excluded from the study. Patients' medical records were then reviewed for information on demographic factors, diagnosis, mechanical ventilation settings, pre-ECMO gas exchange parameters, ECMO technique, lung compliance and chest radiographs. Use of other rescue therapies, renal replacement therapy (RRT), vasopressors and tracheostomy was recorded.

The standard ECMO configuration for support of hypoxemic respiratory failure was VV-ECMO and was configured to deliver 3 to 7 L/minute of cavo-atrial blood flow [23] driven by a centrifugal pump (Maquet-Rotaflow). A low-resistance polymethylpentene oxygenator (Maquet-Quadrox-D) was used for gas exchange. Two circuit connectors were available between the pump head and the oxygenator to provide renal replacement therapy via the ECMO circuit, if required. Heparin was infused unless there was a contra-indication. All cannulae were inserted percutaneously by serial dilation without skin incision and sited using cardiac and vascular ultrasound guidance. When the need for ECMO arose at another hospital, patients were cannulated and stabilized on ECMO by the ICU-based retrieval team at their hospital of origin before being retrieved.

Patients remain in the ICU for the duration of ECMO cannulation and if successfully weaned from ECMO, remain there until they are considered stable for transfer to the ward (no vasopressors, mechanical ventilation or hemofiltration). The ICU consultants continue to monitor patients on the ward who are discharged from ICU, but once patients are discharged from hospital there is no further follow-up and there is no specific ECMO follow-up clinic available in our state. Discharge from the ECMO hospital may occur as a ward bed becomes available at the patient's original center (if the patient was retrieved), or they go home or to a rehabilitation center as considered appropriate by the medical team.

To assess HRQoL, patients were contacted by introductory letter explaining the nature of the study, asking for their consent to participate and informing them that they would be contacted by telephone. A trained assessor, with experience in HRQoL assessment using standardized tools then completed the Short-Form 36 (SF-36) quality of life questionnaire version 2 (QualityMertric Health Outcomes, QualityMetric Incorporated, Lincoln, RI) and the EuroQol EQ-5D [24] (EuroQoL EQ5D, Rotterdam, The Netherlands) by telephone interview, and collected subjective information from the patients on leg weakness and/or numbness, use of ankle foot orthoses, and information about return to work status.

The primary outcome variable was HRQoL measured with the SF-36) [2]. Other outcomes included ECMO-associated complications, survival, discharge destination, HRQoL measured with the EQ-5D [17,24] and return-to-work status. The SF-36 derives a total score from scores in eight domains: physical function, role-physical, bodily pain, general health, vitality, social functioning, role-emotional and mental health. Each item contributes to a separate domain and items are weighted to calculate transformed domain scores, which range from 0 to 100, where 100 represents the best possible health score [25]. Two norm-based component summary scores were also calculated [26]. Normative individual age- and sex-matched Australian population data were used for comparison with transformed domain and component summary scores [27]. The minimum important difference (MID) for SF-36 transformed domain scores has been reported to be at least five points [28]. Consistent with previous intensive care HRQoL studies [2,29], a five-point difference was considered clinically significant.

After a systematic search of the literature and individual correspondence with researchers reporting long term outcomes in ARDS, several international reports of HRQoL (SF-36) were chosen for comparison. These comparators were chosen if they reported HRQoL using the SF-36 at 6 or 12 months after ICU discharge. They were a general ARDS population from Canada [2,20], a population retrieved for ECMO consideration in the UK [17] and an ECMO cohort described during the H1N1 epidemic by the French REVA Study Group [18].

Summary data were collected and expressed as numbers (percentages), normally distributed data reported as means ± SD and non-normal data reported as medians and interquartile range (IQR). For comparisons of SF-36 scores to the US and Canadian [2,20], UK [17], and French populations [18] the Students *t*-test was used. Categorical variables were compared using the chi-square test for equal proportions. Analyses were performed using SAS version 9.2 (SAS Institute Inc., Cary, NC, USA). To reduce the chance of a type I error due to multiple comparisons, a two-sided *P*-value of 0.01 was considered statistically significant.

## Results

**Figure 1:**
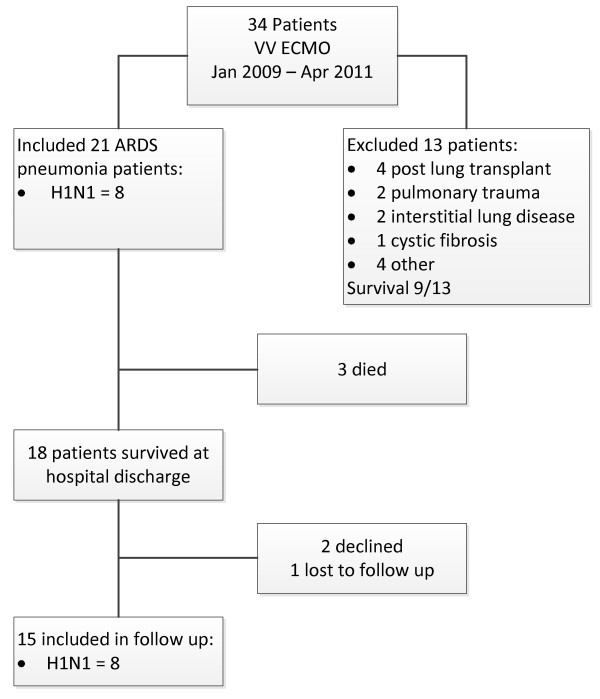
**Flowchart of patients included in the study**. ARDS, acute respiratory distress syndrome; ECMO, extracorporeal membrane oxygenation; H1N1, H1N1 influenza; VV, veno-venous cannulation.

During the 28-month period, 21 adults received ECMO support for refractory hypoxemia due to ARDS (Figure 1). The mean (SD) age was 36.3 ± 12.1 years and 52% of patients were female. Mean body mass index (BMI) was 32.1 ± 10.5 Kg/m^2 ^with seven patients (33%) classified obese (BMI ≥ 30 Kg/m^2^). Demographic data are detailed in Table 1.

**Table 1:** Demographics

Parameter	Result (n = 21)
Age, years, mean ±SD	36.3 ± 12.1
Male sex, n (%)	10 (48)
BMI, mean ± SD	32.1 ± 10.5
ARDS pneumonia, n (%)	21 (100)
Lung injury score, median (IQR) APACHE II, mean ± SD	4 (3.5, 4.0) 19.9 ± 5.8
APACHE II ROD, mean ± SD	33.2 ± 18.1
APACHE III co-morbidity, n (%)* Pregnancy, n (%)	2 (9) 3 (14)
ECMO retrieval, n (%)	18 (86)
H1N1 positive, n (%)	11 (52)

ECMO, extracorporeal membrane oxygenation; n, number; BMI, body mass index; APACHE, Acute Physiology and Chronic Health Evaluation; IQR, interquartile range; ROD, risk of death; *presence or not, of at least one co-morbidity.

The mean APACHE II score on first admission to ICU was 20 ± 6. Eighteen patients (86%) were retrieved from other ICUs by a dedicated ECMO retrieval team; two patients were retrieved from interstate centers, eight from regional centers and eight were from metropolitan hospitals. Eleven patients (52%) had H1N1 influenza A-associated pneumonitis. Three female patients were pregnant at the time of initiation of ECMO.

Conventional ventilation and rescue therapies were used prior to ECMO (Table 2). Median lung compliance on admission to the ICU was 16.6 ml/cmH_2_O (IQR 9.3 to 23.1). All our patients were mechanically ventilated for less than 7 days prior to the initiation of ECMO. The decision to institute ECMO for severe respiratory failure is often complex. In our ICU,, clinical triggers for VV-ECMO initiation include a ratio of the ratio of partial pressure of oxygen to fraction of inspired oxygen (PaO_2 _mmHg/FiO_2_) < 75 (SaO_2 _< 90) hypercapnea with pH < 7.15 with safe mechanical ventilation settings (plateau pressure ≥ 35mmHg and tidal volume ≥ 6 ml/Kg predicted body weight) and extensive (3-4 quadrant) lung infiltrate consistent with acute lung injury despite optimizing circulatory support (cardiac assessment with echocardiography) and inotropes or volume state therapy as appropriate, a trial of high positive end expiratory pressure (PEEP) (18 to 22) and recruitment maneuver (if not contraindicated) and a 2- to 12-hour trial of inhaled nitric oxide or alternative pulmonary vasodilator.

**Table 2:** ICU patient management

Characteristics Severity of ARDS one hour prior to initiation of ECMO	N = 21
Ventilation parameters:	
Pressure control mode of ventilation, n (%) Lowest PaO_2_/FiO_2 _ratio, median (IQR) Highest FiO_2_, median (IQR) Highest PEEP (cmH_2_O), median (IQR) Highest peak pressure (cmH_2_O), median (IQR) Lowest pH, median (IQR) Dynamic lung compliance (ml/ cmH_2_O), median (IQR) Quadrants of X-ray infiltrate (n), median (IQR)	21 (100) 69 (50, 105) 1.0 (1.0, 1.0) 17 (15, 20) 30 (27, 33) 7.2 (7.1, 7.3) 16.6 (9.3, 23.1) 4 (4-4)
Rescue therapies, n (%)	
Recruitment maneuver Nitric oxide HFOV	16 (76) 3 (14) 1 (5)

**After initiation of ECMO**	

Vasopressor, n (%)	16 (76)
Renal replacement therapy, n (%)	2 (10)
ECMO parameters	
Converted dual flow, n (%) High blood flow (L/min), mean ± SD High FGF, mean ± SD Highest platelets, mean ± SD Highest Hb, mean ± SD	14 (67) 5.2 ± 1.0 5.3 ± 1.9 219 ± 114 90.2 ± 12.7

ARDS, acute respiratory distress syndrome; ECMO, extra corporeal membrane oxygenation; FGF, free flow gas; Hb, hemoglobin; HFOV, high frequency oscillatory ventilation; IQR, interquartile range; n, number; PEEP, positive end expiratory pressure.

Eighteen patients (86%) (Table 3) survived to hospital discharge and at least 17 of these people were still alive at the time of follow-up, with one lost to follow-up. All three pregnant women and one fetus survived. Three patients -(14%) died in the ICU due to the underlying severe disease process, including one patient with severe sepsis, one with multiple organ failure and one with severe intrapulmonary haemorrhage. ECMO was provided for a median of 10.6 (3.6 to 15.8) days and mechanical ventilation for a median of 15.3 (12.0 to 23.2) days from admission to the ECMO center (Table 3). Length of stay (LOS) at the ECMO center was highly variable with an ICU LOS of 20.7 (14.9 to 28.6) days and ECMO center hospital LOS of 28.4 (18.5 to 37.7) days (Table 3). Of the eighteen survivors, eight (44%) were discharged directly home. Nine (50%) were discharged to another acute hospital, including three (17%) who transferred to another ICU, and one (6%) who transferred to an inpatient rehabilitation facility. Only 12 survivors (67%) were ambulant at discharge. No patients described ongoing problems as a result of ECMO cannulation.

**Table 3:** Outcome Measures

Outcome measure	N = 21
ICU outcomes	
ICU length of stay, days, median (IQR)	20.7 (14.9, 28.6)
Duration of mechanical ventilation, days, median (IQR)	15.3 (12.0, -23.2)
Duration of ECMO support, days, median (IQR)	10.6 (3.6, 15.8)
Survival at ICU discharge, n (%)	18 (86)
Reintubation, n (%)	1 (5)
Tracheostomy, n (%)	12 (57)
Pressure areas, n (%)	17 (81)
Hospital outcomes	
Hospital length of stay, days, median (IQR)	28.4 (18.5, 37.7)
Survival at hospital discharge, n (%)	18 (86)
Cause of death, n (% of deaths)	3 (14)
Intrapulmonary hemorrhage Sepsis Multiple organ failure	1 (5) 1 (5) 1 (5)
**Outcome of survivors** Days to follow-up, median (IQR)	**N = 18** 261 (225, 571)
Ambulant at hospital discharge, n (%)	12 (67)
Discharge destination, n (%)	
Home Other hospital Rehabilitation facility	8 (44) 9 (50) 1 (6)
Returned to work (n = 15) Returned to original work (n = 15)	8 (53%) 4 (26%)

ECMO, extra corporeal membrane oxygenation; IQR, interquartile range; N, number.

Comparison with two other large VV-ECMO series [15,17] (Table 4), show non-statistically significant differences in baseline variables, and a shorter LOS and lower mortality rate in our cohort. Mortality was similar to other recent Australian ECMO data [30] and has improved at our center over the past 3 years (hospital survival at our center for ARDS from 2003 to 2008 was 62% of 13 patients, compared to the period from 2009 to 2011, in which it was 86%).

**Table 4:** Comparison of ARDS populations reported as mean ± SD or median (IQR)

	Current study	**ECMO Group UK study **[17]	**REVA **[28]	**Non-ECMO ARDS Canada **[20]
Follow-up, months	8	6	12	6
Number	21	90	12	117
Age, years	36 ± 12	40 ± 13	36 (30, 39)	45 (36, 58)
APACHE II	20 ± 6	20 ± 6	n/a	23 (17, 27)
Pneumonia (%)	100	62	100	53
ICU LOS, days	21 (15, 29)	24 (13, 41)	38 (19, 67)	25 (15, 45)
Hospital LOS, median days	28 (15, 29)	35 (16, 74)		48 (27, 77)
ECMO, %	100	76	100	n/a
Death at 6 months, n/study population (%)	3/21 (14)	33/90 (37)	24/67 (36)	78/196 (40)
Lowest PaO_2_/FiO_2 _ratio (day 1)	80 ± 40	76 ± 30	< 50*	< 200*
PEEP	15 ± 4.7	13.7 ± 9.6	≥ 5*	n/a

Results are presented as mean ± SD or median (interquartile range, IQR) unless stated otherwise. ARDS, acute respiratory distress syndrome; ECMO, extracorporeal membrane oxygenation; LOS, length of stay; N, numbers of patients included in the study; n/a, not available; PaO_2_/FiO_2_, ratio of partial pressure of oxygen to fraction of inspired oxygen; PEEP, positive end expiratory pressure; *study inclusion criteria.

Fifteen survivors (83% of 18 hospital survivors) consented via telephone to long-term follow-up and were evaluated for long-term outcomes and quality of life. Two refused to consent to follow-up and one was lost to follow-up. Of these fifteen patients, eight were PCR-positive for H1N1 on nasopharangeal swab, and seven had ARDS due to other causes of pneumonia. All described bilateral limb weakness (ICU-acquired weakness) that continued beyond discharge, and one patient had severe myopathy and polyneuropathy with ongoing disability that required a splint for the management of foot drop. Seventeen patients (81%) developed pressure injuries during their stay. No patients required ongoing use of oxygen after primary hospital discharge. Although eight survivors (52%) had returned to work, four (26%) of this population had returned to previous work levels at the time of follow-up.

Sequelae and HRQoL were evaluated for the 15 long-term survivors. Median follow-up was 8.4 (6 to 16) months (Table 4). One patient was unable to be contacted for a prolonged period (16 months) due to a long visit overseas. There were no significant differences between patients in the study with ARDS as a result of H1N1 influenza compared to ARDS, due to non-viral pneumonia for any single domain of the SF-36 or for the physical component summary, although the numbers for this comparison were very small. The mental component summary score was lower for the H1N1 patients compared to non-viral pneumonia (mean 28.8 ± 13.3 versus 44.1 ± 13.4, *P *= 0.04).

**Figure 2:**
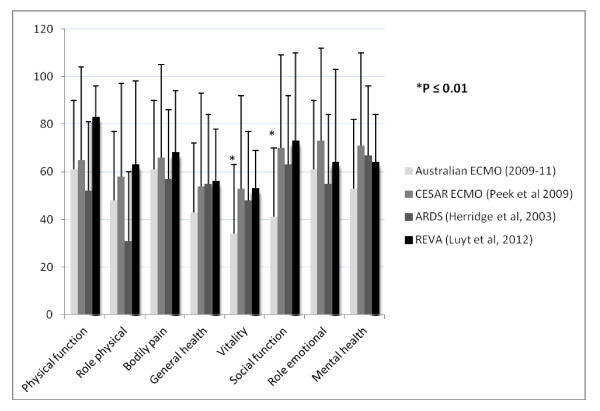
**Comparison of adult acute respiratory distress syndrome (ARDS) survivors from different populations for Short-Form (SF)-36 quality of life (QoL)**. ECMO, extracorporeal membrane oxygenation; Australian ECMO, current ECMO cohort; REVA, Research in Mechanical Ventilation (*Réseau européen de recherche en Ventilation Artificielle*).

Mean SF-36 scores were significantly lower (*P* < 0.05) in patients who had received ECMO than in matched healthy controls for all domains of the SF-36 except bodily pain and role-emotional. This current study cohort of long-term ARDS survivors receiving ECMO support had similar scores in the domains of physical function, physical role, bodily pain, general health, role-emotional and mental health, compared to both comparator ARDS populations (Figure 2) but they had significantly lower scores in the domains of social function and vitality (Figure 2).

Quality of life was also reported using the EQ-5D (Table 5). In this cohort, five survivors (42%) were unable to perform usual activities and described severe or extreme anxiety and depression. Interestingly, 57% of patients were ambulant at hospital discharge but using the EQ5D, 53% of survivors reported slight to severe problems with mobility at follow-up. The majority of survivors had no problems with personal care (80%). There was no significant difference in overall health status (EQ-5D visual analogue scale 0 to 100) when comparing our cohort with the UK CESAR study.

**Table 5:** Results of EQ-5D quality of life for The Alfred ARDS survivors who received VV-ECMO: comparing to other ECMO survivors

EQ5D (English v.2 © 2010 EuroQol Group. EQ-5D™ version for Australia)	Current study cohort, n = 15 (EQ5D v2, five-point scale)
Problems with mobility:	
None Slight Moderate Severe Unable	7 (47%) 6 (40%) 1 (7%) 1 (7%) 0
Problems with personal care (washing/dressing):	
None Slight Moderate Severe Unable	12 (80%) 2 (13%) 1 (7%) 0 0
Problems with usual activities:	
None Slight Moderate Severe Unable	6 (40%) 2 (13%) 2 (13%) 0 5 (42%)
Pain/discomfort:	
None Slight Moderate Severe Extreme	3 (25%) 5 (42%) 5 (42%) 2 (13%) 0
Anxiety/depression	
None Slight Moderate Severe Extreme	5 (42%) 2 (13%) 3 (25%) 3 (25%) 2 (13%)
Overall health status (VAS, 0 to 100) mean ± SD	65.9 ± 18.6

Results are presented as number (%) unless otherwise stated. ARDS, acute respiratory distress syndrome; ECMO, extracorporeal membrane oxygenation; N, number; VAS, visual analogue scale; v1, version 1; v2, version 2; VV, veno-venous.

## Discussion

### Key findings

This was a small, single-center long-term follow-up study of young patients with ARDS, mainly related to H1N1 influenza A pneumonitis, who received rescue VV-ECMO. The survival rate was similar to other Australian published data [30], but was higher than in international ECMO cohorts. While these patients had a high incidence of pressure injuries and long stays in the ICU and hospital, the LOS was shorter than comparable ARDS cohorts [20] and many were discharged directly home. The HRQoL in survivors was significantly less than normal age- and sex-matched Australian people, with physical and mental limitations at the time of follow-up. The SF-36 domains of social function and vitality were also reduced compared to previously reported survivors of ARDS [20] and other ECMO series [17,18]. Only a quarter of patients had returned to previous work at the time of follow-up, which is similar to previously described ARDS cohorts [20].

### Relationship to previous studies

On long-term follow-up, our cohort had substantial physical limitations compared with Australian age- and sex-matched samples. Their SF-36 physical component score was 20% lower than normal, with severe limitations in the domains of physical function, role-physical, social function and mental health, general health and vitality. They also displayed reduced mental wellbeing with their SF-36 mental component score 27% lower than normal. This was more pronounced in the patients with ARDS resulting from H1N1, compared to patients with ARDS pneumonia, and may be caused by the systemic effects of H1N1 virus, rather than differences in ICU management, such as the use of sedatives. This indicates frequent psychological distress and social and role disability due to emotional problems. ARDS survivors have been previously described as having considerable challenges, including reduced exercise capacity, cognitive dysfunction and depression or post-traumatic stress disorder (PTSD) [31-33].

Compared to other groups of ARDS survivors in the literature, HRQoL was statistically reduced in this cohort of patients in the domains of vitality and social function (Table 5) [17,20]. However, consistent with previous intensive care HRQoL studies [2,29], a five-point difference in SF-36 transformed scores was considered clinically significant. When this definition of clinical significance was applied, this cohort had clinically reduced domains of general health, vitality, social function, and mental health.

Vitality is a lack of fatigue, or feelings of energy, and social function is the degree to which relationships are maintained with friends and family. It is unclear why our cohort would have reduced energy or inability to maintain social relationships compared to other survivors of ARDS. Importantly, only half of this young cohort had returned to work and a quarter of them had returned to previous work levels. Previous studies have reported an important functional association with survivors of ARDS that have moderate-severe depression symptoms and are less likely to have returned to work compared to those with less severe symptoms [34]. Depression has been reported in up to 50% of ICU survivors 12 months after discharge [35]. Psychiatric screening is also very important to assess depression and PTSD, which is prevalent in ICU survivors [2,29]. However, compared with the French H1N1 cohort who received ECMO [18], our patients who received ECMO had reduced HRQoL in both domains of vitality and social function at a median of 8 months follow-up. The study by Luyt *et al*. (2012)[18] found no difference between survivors of severe H1N1 who received ECMO and those who did not when assessed at 12 months after the stay in ICU. This important question needs to be further addressed in an Australian population comparing survivors of ARDS who do or do not receive ECMO at the same time point.

Compared to other published series of patients with ARDS receiving ECMO for refractory hypoxemia [15,17,18], our small cohort appeared to have good survival rates. When compared with the UK ECMO study [17] this difference was not accounted for by illness severity (Apache II, lowest PaO_2_/FiO_2 _ratio on day 1), nor patient age, which were similar in both series (Table 4). It may have been accounted for by the much higher incidence of influenza A pneumonia in our cohort but when the 56 patients with H1N1 pneumonia from the UK ECMO study were separately analysed, the mortality was still significantly higher than in our cohort (27.5 versus 14.0%) [36]. Several factors that may have accounted for better outcomes included increased management of viral pneumonitis due to the H1N1 epidemic, improved ambulance retrieval service, the ECMO technology, including ultrasound-guided percutaneous ECMO cannulation, intensivist-driven care from retrieval to decannulation and extensive ECMO experience (10 years) at the center.

Patients were retrieved with severe life-threatening refractory hypoxemia if they did not respond to conventional rescue therapies. These included inhaled nitric oxide and recruitment maneuvers, which are consistent with previously published data [19] from Australia where the most common rescue therapies used during the H1N1 epidemic were recruitment maneuvers (67%) and inhaled nitric oxide (32%) [19]. In the majority of patients in both this study (86%) and the UK ECMO study (69%), the need for ECMO arose in an external hospital and the patients were retrieved to the study center hospital. In the UK ECMO study [17] all such retrievals were performed on conventional ventilation and ECMO was initiated on arrival at the study center hospital after transfer. The UK study described three deaths before the retrieval team reached the initial hospital and two deaths in transit.

ECMO was maintained in this cohort for a long period of time in comparison to other studies of patients receiving ECMO for severe ARDS [15,17]. Despite the long duration of ECMO, our cohort had a shorter ICU and hospital LOS at the ECMO center (Table 4) compared to the group of patients with ARDS receiving ECMO [17] or ARDS without ECMO [20]. This may be partly explained because a large portion of our cohort (50%) was discharged to other acute-care facilities, mostly the destination from which they were retrieved. It may also be a result of the fact that a large proportion of our cohort had confirmed H1N1. Recent data from the H1N1 registry of the Extracorporeal Life Support Organization (ELSO) [37] showed 61% survival from 76 international centers of H1N1 requiring ECMO, including adult and pediatric data.

A large number of patients in the current study suffered from pressure injuries probably due to prolonged immobility with no long-term effects as a result. This has led to a change in practice in the ICU that includes decreased time between turning patients receiving ECMO, air mattresses and mechanically rotating beds. One patient died after developing a pneumothorax prior to ECMO, then suffering a severe pulmonary hemorrhage on insertion of an intercostal catheter once ECMO and anticoagulation therapy had been commenced. One survivor suffered severe ICU-acquired weakness, was discharged to a rehabilitation facility and was required to wear a splint long-term for foot drop. No patients required ongoing domiciliary oxygen.

Improved survival with decreased HRQoL places a significant burden on caregivers, patients and infrastructure. Survivors have profound muscle weakness and wasting [20], which impairs exercise capacity and may be improved with early rehabilitation during the ICU period [38,39] and a prolonged hospital stay, which includes inpatient rehabilitation. More than half of our survivors reported some degree of problems with mobility at follow-up (Table 5). Further research is required to establish the physical outcomes, exercise capacity and rehabilitation requirements of survivors and to identify risk factors that predict a poorer HRQoL [31,32].

### Implications of study findings

In this cohort, many patients suffered from lack of vitality and social function and reported feelings of isolation on discharge from hospital. In particular, most of this cohort did not return to previous work, which may contribute to their poor social function and is different to other reports of ECMO survivors [18]. ICU outpatient review of these patients may be required to address functional limitations in survivors. While ECMO survivors had reduced HRQoL, it remains unclear whether the complications of ECMO played a role in this outcome. Future prospective studies are required to confirm these findings and are planned as part of a multi-center trial investigating outcomes of survival of ARDS patients receiving ECMO.

### Limitations

This pilot study has a number of limitations. First, our major limitation is the small number of included patients, despite collection during a viral pandemic, which limits the external validity of the results. Second, owing to the nature of the pandemic, there were no injury-matched controls (young, previously well patients without co-morbidities all received ECMO as a rescue therapy if they were severely hypoxemic). Third, the data set was examined retrospectively, and therefore the follow-up period for HRQoL was variable, which limits the comparison to other studies. Fourth, there is limited information on the issues contributing to a decrease in HRQoL as a result of the telephone interview process, with no in-person follow-up, although previous work examining telephone interviews has shown that the response rate is improved but patients may report more favourable health ratings [40]. Finally, we aimed to compare our results to international data that have inherent differences in the structure and provision of the ECMO service, or the management of ARDS, which may confound the results. Future work in this area, where possible, should include larger numbers with a comparator group without ECMO from the same population.

## Conclusions

The high survival rate of patients receiving ECMO for severe ARDS in Australia was associated with reduced HRQoL compared to the normal population, and also compared with other ARDS survivors, particularly in the SF-36 domains of vitality and social function. Few of the survivors returned to previous work levels. The results highlight the importance of long-term follow-up and support of patients on discharge from the ICU and emphasize that traditional short-term endpoints in clinical studies do not always reflect long-term outcomes [41].

## Key messages

• Health related quality of life was reduced in the domains of vitality and social function.

• The majority of patients described ongoing depression and anxiety that was moderate to extreme at follow-up.

• Only a quarter had returned to their previous work levels at follow-up.

## Abbreviations

ARDS: acute respiratory distress syndrome; ECMO: extracorporeal membrane oxygenation; EQ-5D: EuroQol health related quality of life questionnaire; HRQoL: health related quality of life; IQR: interquartile range; LOS: length of stay; n/a: not available; MID: minimum important difference; PaO_2_/FiO_2_: ratio of partial pressure of oxygen to fraction of inspired oxygen; PCR: polymerase chain reaction; PEEP: positive end expiratory pressure; RRT: renal replacement therapy; SF-36v2: Short Form-36 version 2.

## Competing interests

The authors declare that they have no competing interests.

## Authors' contributions

CH and KH designed the study, co-ordinated and drafted the manuscript with VP, DJC, TE and AD. CH and TE completed data extraction and interviews. Data analysis was conducted by CH and MB. AN and DT gave expert content to the manuscript. All authors read and approved the final manuscript.
